# Prospective multicenter observational trial on the safety and efficacy of LEVORAG^®^ Emulgel in the treatment of acute and chronic anal fissure

**DOI:** 10.1007/s10151-015-1289-2

**Published:** 2015-03-15

**Authors:** R. Digennaro, G. Pecorella, S. La Manna, A. Alderisio, A. Alderisio, B. De Pascalis, D. Pennisi, G. Santangelo, F. Pezzolla, A. Racalbuto, G. Serra, A. Pulvirenti D’Urso, D. F. Altomare

**Affiliations:** 1 Department of Emergency and Organ Transplantation, University of Bari, Piazza G Cesare, 11, 70124 Bari, Italy; 2 Department of Surgery and Oncology, Vittorio Emanuele Hospital, Catania, Italy; 3 Department of General and Oncologic Surgery, University of Naples, Naples, Italy; 4 Coloproctological Unit Azienda Ospedaliera “S. Anna e S. Sebastiano” di Caserta, Caserta, Italy; 5 Department of General Surgery, Ospedale Ignazio Veris Delli Ponti, Scorrano, Italy; 6 Department of Surgery, IRCCS De Bellis, Castellana Grotte, Bari, Italy; 7 Department of General Surgery, University of Catania, Policlinico G. Rodolico, Catania, Italy; 8 Department of General Surgery, Brotzu Hospital, Cagliari, Italy; 9 Colonproctology Unit, Garibaldi-Nesima Hospital, University of Catania, Catania, Italy

**Keywords:** Anal fissure, Emulgel cream, Chemical sphincterotomy

## Abstract

**Background:**

Anal fissure (AF) is a common cause of anal pain with a tendency not to heal spontaneously because of ischemia of the anoderm caused by sphincter spasm. Lateral internal sphincterotomy, while very effective, can cause fecal incontinence and chemical sphincterotomy by application of cream may have discouraging side effects and/or low efficacy. The aim of this prospective multicenter study was to evaluate the safety and effectiveness of a new medical treatment based on Emulgel cream, with emollient, soothing and protective agents, on AF healing.

**Methods:**

Consecutive patients with AF treated in nine coloproctology units during 6 months entered the study on topical treatment with Levorag^®^ Emulgel (THD S.p.A Correggio (RE), Italy). Before treatment, they had a proctologic examination and pain was measured using a visual analog scale. THD Levorag^®^ Emulgel was applied every 12 h for 40 days. Monitoring was scheduled at 10, 20 and 40 days. At time 0 and at the end of treatment, patients underwent anorectal manometry, if possible.

**Results:**

Two hundred eighty-four AF patients were recruited (171 acute fissures). Complete healing was achieved in 47.9 % of the cases, an improvement in 31.0 % (global efficacy 78.9 %). In patients with acute fissure, the rate of efficacy was 89.4 % (complete healing: 64.3 %, improvement: 25.1 %), in those with chronic fissure the rate of efficacy was 62.8 % (complete healing: 23 %, improvement: 39.8 %), *p* < 0.001. Pain and resting anal pressure decreased significantly after treatment.

**Conclusions:**

Treatment with THD Levorag^®^ Emulgel proved to be effective for the reepithelization of AF and the reduction of pain in the short term in about 80 % of patients.

## Introduction

Anal fissure (AF) is one of the most common anorectal disorders, and, in Italy, the second most common problem, after hemorrhoids, that brings patients to the proctologist’s office [1]; AF is responsible for intense and prolonged anal pain, often fails to respond to analgesic treatment with consequent deterioration of the patient’s quality of life [2, 3].

AF is characterized by “a linear ulcer of the anoderm, distal to the dentate line” [4], is usually caused by minor anal trauma at defecation and accounts for about 40 % of the annual visits to a coloproctology unit according to a recent annual report of the Italian Society of Colorectal Surgery [1].

Despite significant progress made both in understanding the etiology and pathogenesis of AF, and in developing treatments based on internal sphincter relaxant agents [5], there is still debate about what constitutes optimal treatment is. In fact, AF treatment is one of the issues most frequently studied and documented in the literature, which is an indication of the clinical relevance of the problem with and the awareness that the available treatment options are still unsatisfactory.

The topical myorelaxant creams on the market were shown to have limited advantages when compared with placebo in randomized controlled trial [6, 7] and significantly lower efficacy compared to lateral internal sphincterotomy (LIS) [8], while the option of using botulinum toxin A injection is being abandoned [9, 10].

Furthermore, most of the myorelaxant creams available (containing nitric oxide donors) have some unpleasant side effects like headache, migraine and *pruritus ani* [7, 11], which affect patients’ compliance, while other myorelaxants with few side effects (calcium channel blockers) are not available in our country. On the other hand, LIS, which is necessary in about one-third of AF patients [1] can rapidly relieve symptoms, but the risk of early or late fecal incontinence [12], even if low, prevents its application as a first line therapy.

The availability of new effective and well-accepted topical treatment, without side effects, is therefore eagerly awaited.

The aim of our prospective multicenter observational study was to evaluate the safety and efficacy of a new topical cream, Levorag^®^ Emulgel (THD, SpA, Correggio, (RE), Italy), in reducing pain and in the reepithelization of AF.

## Materials and methods

All consecutive patients with acute or chronic anal fissure examined and treated in nine coloproctology units in Southern Italy between June 2013 and January 2014, who gave their informed consent to the study, entered this prospective observational trial which qualified for “exempt” on institutional review board because it was part of the routine internal audit of the colorectal units involved in the study.

Inclusion criteria were primary AF, acute or chronic disease, and age range between 18 and 65 years. Acute fissure was defined as a recent (within 1 month) ulceration of the anoderm without the typical signs of a chronic fissure (sentinel tag, induration of the lateral margins of the fissure, eventual exposure of the internal anal sphincter) [6]. Patients already under treatment with other topical creams (containing nitric oxide donors, or calcium channel blockers), as well as those with constipation requiring prolonged straining or manual maneuvers during evacuation, fecal incontinence, neoplasms, human immunodeficiency virus infection, insulin dependent diabetes mellitus, abscess and anal fistula, Crohn’s disease, advanced liver disease, and pregnancy were excluded from the study.

The treatment included a topical application every 12 h of THD Levorag^®^ Emulgel for 40 days. THD Levorag^®^ Emulgel is a gel cream in 3.5-ml single-dose tubes containing a mixture of substances with lubricating, film-forming, emollient, soothing and protective properties (Table 1). In particular, the lubricating agents were included to create a film on the AF thus facilitating stool expulsion and favoring pain reduction. Hydrolyzed hibiscus esculentus extract was added to improve the elasticity of the tissues which, in combination with the expected decrease of anal pain, according to the manufacturer’s instructions should lower the resting anal pressure, improving the microperfusion of the anoderm. Finally, the sodium carboxymethyl beta-glucan component was added to favor the reepithelization process.

**Table 1:** Active components of THD Levorag^®^ Emulgel

Active components of THD Levorag^®^ Emulgel
Hibiscus esculentus extract
Carboxymethyl beta-glucan
Dimethicone, glycerine, prunus amygdalus dulcis oil, borago officinalis seed oil
Malva sylvestris extract, calendula officinalis extract, glycyrrhiza glabra extract

The patients were invited to apply the creame with the tip of the finger instead of inserting the anal cannula included in the package because the use of the applicator (4.5 cm long) will deliver the gel to the rectum instead of the anal canal. The patients were also invited to take oral laxatives in order to reduce the hardness of the stools and facilitate evacuation, and to clean their anus with disinfecting chlorhexidine-based solutions (Fisian, VALDERMA srl, Terranuova Bracciolini, Italy). The use of oral painkillers was allowed and recorded.

Clinical evaluation of patients was scheduled at time 0, before starting the treatment, and at days 10, 20 and 40. Baseline evaluation included a proctologic exam to determine the diagnosis and the type of fissure, and recording the amount of anal pain, using a visual analog scale (VAS), and other concomitant anal symptoms. Anal manometry to document the anal resting pressure before and after treatment was carried out in two of the units involved in the study.

On day ten of treatment, the patients were interviewed by phone to evaluate the level of pain and those without any improvement or with further deterioration of symptoms were dropped from the study.

On day 20 of treatment, the patients underwent a proctologic examination and pain was assessed with a VAS. The degree of reepithelization of the fissure was scored as follows: 0: deep fissure still present; 1: superficial fissure; 2: partial reepithelization or 3: complete healing and reepithelization. Those without any improvement in reepithelization or pain score were dropped from the study.

On day 40, the remaining patients underwent their last evaluation including a proctologic examination, anal pain score, and, if possible, anal manometry.

The results were divided according to outcome (successful treatment and failure). Successful treatment was defined as complete fissure healing or partial fissure reepithelization associated with at least 50 % reduction of the pain score after completing the scheduled 40 days of treatment.

### Sample size calculation

Sample size calculation were carried out by the website http://www.raosoft.com/samplesize.html.

Assuming the VAS score would be reduced to 50 % of the initial value, with an alpha error of 5 % and a beta error of 10 %, and considering a possible dropout rate of 5 %, a total of 281 patients were required to get statistical significance with a 90 % confidence interval and a power of the study of 90 %.

### Statistical analysis

All data were prospectively entered into an excel database by each of the participating centers and analyzed by the coordinating center. Qualitative data were expressed using the Wilcoxon rank sum test for paired data, while median anal manometry data and pain scores were analyzed with the Chi-square test. The ANOVA for repeated measures was performed to evaluate the changes of pain scores over time. A *p* value <0.05 was considered statistically significant.

## Results

Overall, 284 patients (130 males, mean age 44 ± 15 years) were recruited in the nine colorectal units. One hundred twenty-one of them had chronic AF, and 171 had acute AF. The site of the fissure was posterior in 212 cases (74.7 %), anterior in 58 (20.4 %) and anterior and posterior in 14 (4.9 %). The median anal pain score at baseline was 8 on a 0–10 VAS (10 = worst possible pain), while anal manometry revealed high resting pressure (median 95 mmHg) in all the 50 patients examined. A similar baseline average pain score was observed in patients with acute or chronic AF (8 vs 7.7 respectively, *p* = 0.8).

At day 10 of treatment (T1), six patients interviewed by phone (1 with acute fissure) were excluded from the study because of no pain relief. One more patient (with acute fissure) was lost to follow-up.

At day 20 (T2) after clinical examination, another patient (with acute fissure) was lost to follow-up and 11 patients (four with acute fissure) were excluded from the study (because of unaltered persisting pain.

**Fig. 1:**
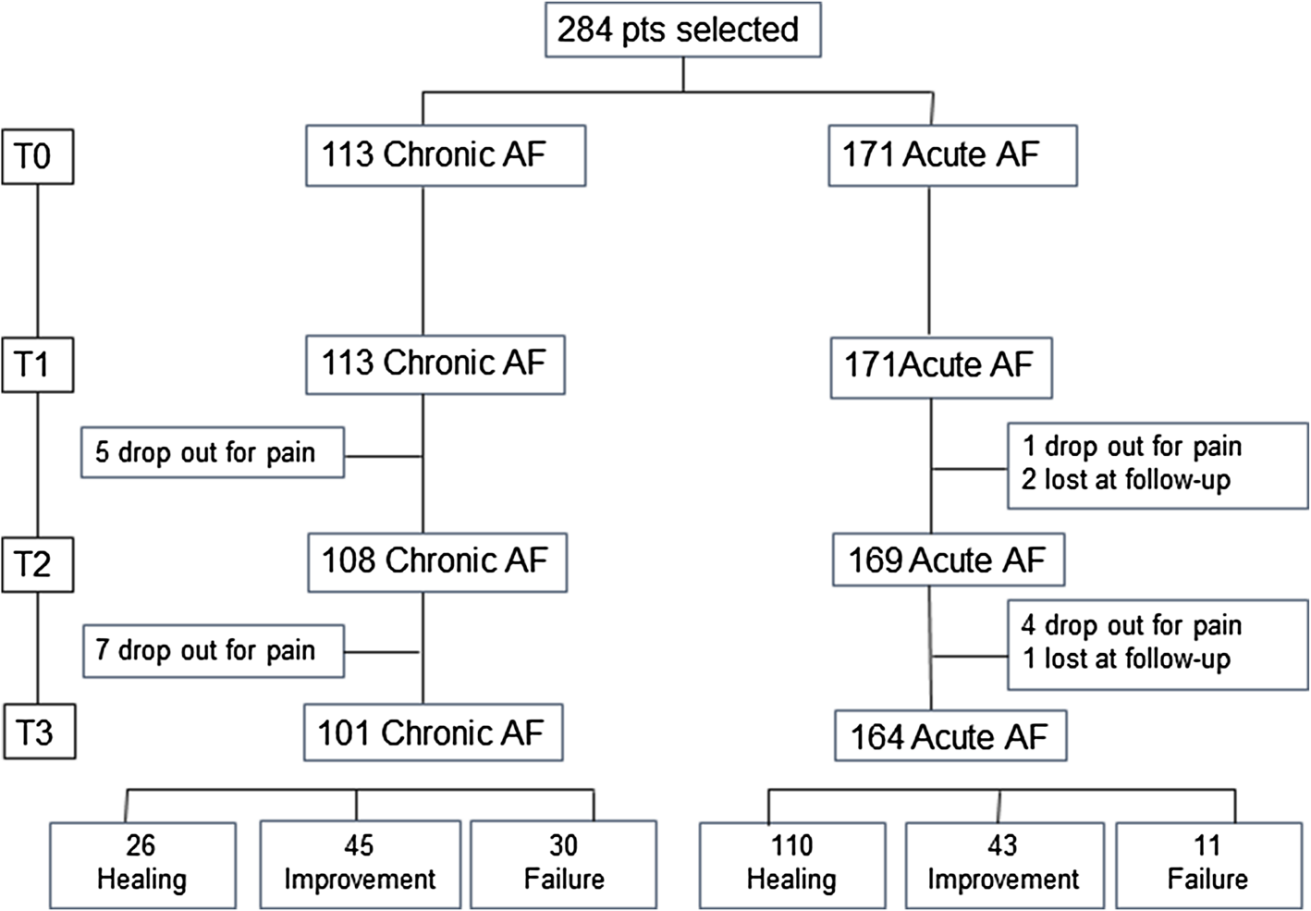
Flow chart showing the outcome of the trial

At day 40, a total of 265 patients were available for final evaluation, 164 with acute fissure and 101 with chronic fissure. Overall, complete healing was achieved in 47.9 % of the cases, and an improvement in 31 % (global efficacy of 78.9 %). In patients with acute fissure, the rate of efficacy was 89.4 % (complete healing: 64.3 %, improvement: 25.1 %), while in those with chronic fissure, it was 62.8 % (complete healing: 23 %, improvement: 39.8 %) (*p* < 0.005) (Fig. 1).

**Fig. 2:**
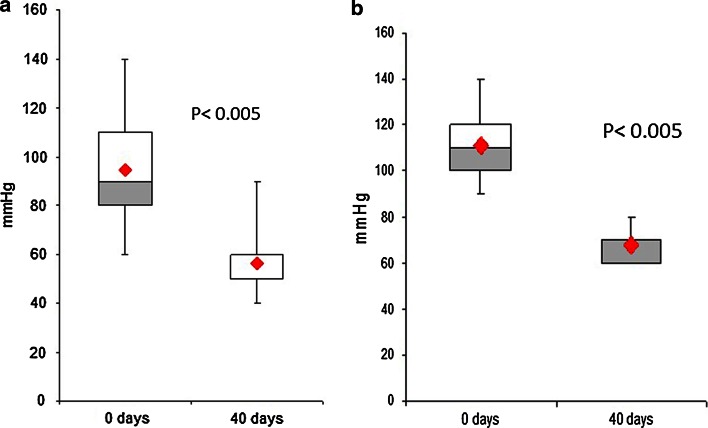
**a** Anal manometry before and after 40 days of treatment in patients with acute anal fissures. **b** Anal manometry before and after 40 days of treatment in patients with chronic anal fissures

The resting anal pressure in the 50 patients who underwent anal manometry decreased significantly after treatment, from a median of 95 mmHg at baseline to 60 mmHg (*p* < 0.005), corresponding to a 40 % reduction (Fig. 2).

**Fig. 3:**
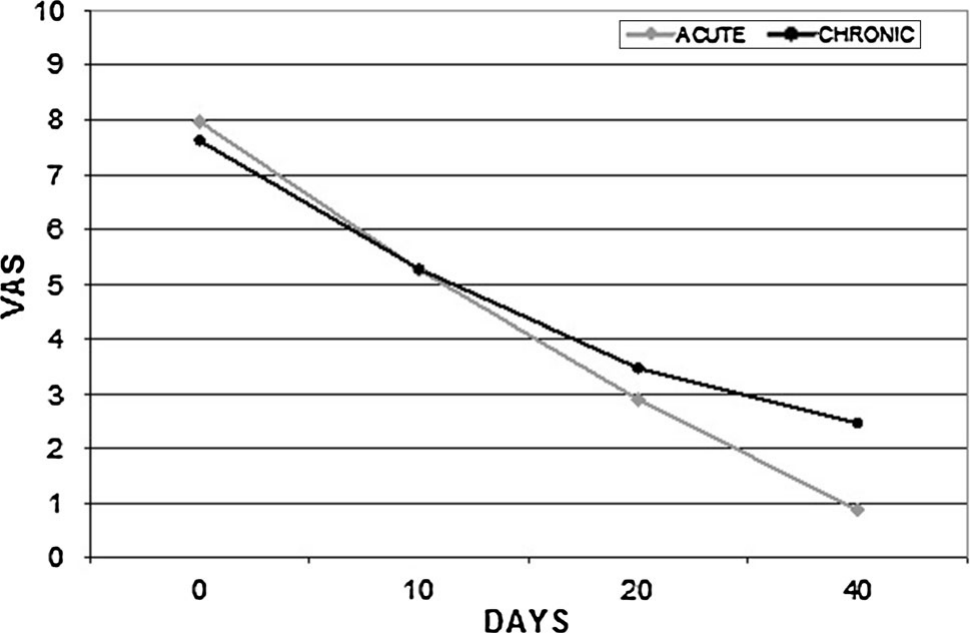
Pain score changes over time, in patients with acute and chronic anal fissure (*p* < 0.001, ANOVA)

The analysis of the VAS score for pain in the group of patients who completed the study showed a progressive and constant lessening of pain and a reduction of 81 % at the end of the treatment (*p* < 0.001), but without a significant difference between the acute and chronic AF (Fig. 3).

**Fig. 4:**
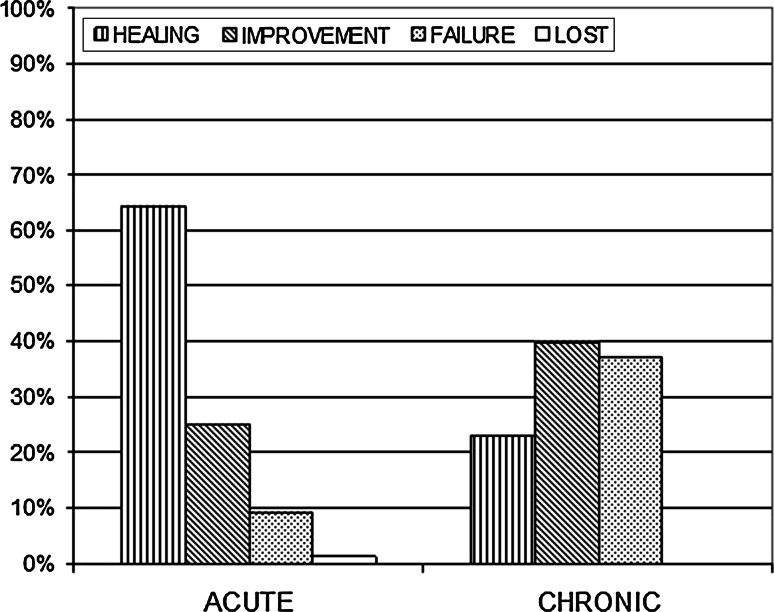
Outcome of conservative treatment with Levorag^®^ Emulgel in acute or chronic anal fissures (*p* < 0.001)

No difference in the healing rate was observed according to the sex of the patients (*p* = 0.58) or the site of the fissure, (*p* = 0.77) while, as expected, acute fissure were more likely to heal than chronic fissures (89.4 and 62.8 %, respectively, *p* < 0.001) (Fig. 4).

Minor adverse effects consisting of soiling and an unpleasant smell due to the cream were reported by 6 % of the patients but in no cases led to changes in the adherence to the scheduled treatment.

Eighty-eight patients, who improved but did not have complete fissure healing at the end of the study period, spontaneously prolonged the treatment with THD Levorag^®^ Emulgel out of the protocol for further 20 days. Thirteen of them (14.8 %) finally achieved complete healing. Lateral internal sphincterotomy was performed on 72 % of the patients who failed with conservative treatment (data available only from three of the nine centers).

## Discussion

After the pathogenic mechanisms underlying the tendency of AF not to heal spontaneously were clearly described by Schouten and co-workers [13] and by Lund and co-workers [14], treatment of AF with chemical sphincterotomy using myorelaxant creams, and thus avoiding the risk of iatrogenic anal incontinence, has become a major goal in proctology. A recent Cochrane review [7] on this topic concluded that no medical treatment could claim the efficacy of surgical sphincterotomy when compared in prospective randomized controlled trial and that, among the medical treatment examined, only glyceryl trinitrate ointment showed a marginally significant advantage compared to placebo. Furthermore, a significant proportion of these patients drop out of treatment because of headache, and up to 50 % of them experience symptom recurrence in the long term [7].

The need of further medical solutions to this painful disease has therefore been eagerly awaited.

Although the lack of a control group is an important limitation to our study, this large multicenter prospective trial shows that topical treatment with THD^®^ Levorag^®^ Emulgel can help about 89 % of patients with acute AF and 63 % of those with chronic AF, leading to complete fissure healing in 64.3 and 23 % of acute and chronic AF, respectively, and allowing a significant reduction of anal pain after the first week of treatment and in the following weeks. Moreover, a further percentage (about 15 %) of fissures not yet healed within the study period completed the healing process in the next 20 days of treatment. The adoption of strict criteria for defining fissure healing, used for the first time in this study, made the results of treatment even more reliable. Another limitation of this study is that it was explicitly designed to evaluate the ability to control the anal pain which is the major patient complaint, and therefore, the sample size was calculated based on this instead of the healing rate.

The association of substances promoting tissue healing contained in THD Levorag^®^ Emulgel resulted in a good patient compliance and no side effects, while the reduction of the anal resting tone was documented in a significant subgroup of these patients.

## Conclusions

THD Levorag^®^ Emulgel was proven to be an effective and well-tolerated topical treatment for both acute and chronic AF in the short term, constituting one more arrow to the bow of proctologists in treating this painful disease, and compares well with the other conservative medical treatments even if the long-term outcome remains to be evaluated.
